# Proposition of Cutoff Points for Anthropometric Indicators to Identify High Blood Pressure in Adolescents

**DOI:** 10.3389/fnut.2022.874047

**Published:** 2022-07-18

**Authors:** Leandro Lima Borges, Aline Mendes Gerage, Luciana Zaranza Monteiro, Anderson Zampier Ulbrich, Diego Augusto Santos Silva

**Affiliations:** ^1^Sports Center, Federal University Santa Catarina, Florianópolis, Brazil; ^2^University Center of “Distrito Federal”, Brasília, Brazil; ^3^Federal University of Parana, Curitiba, Brazil

**Keywords:** blood pressure, anthropometry, accuracy, youth, cutoff points

## Abstract

**Aim:**

To propose cutoff points for anthropometric indicators for high blood pressure (HBP) screening in adolescents and to identify, among these indicators, those more accurately for boys and girls.

**Methods:**

This cross-sectional study was carried out in the city of São José, SC, Brazil with 634 adolescents aged 14 to 19 years. Blood pressure levels were measured using a digital oscillometric sphygmomanometer and adolescents were classified as having HBP or not. Anthropometric indicators were calculated based on anthropometric measurements such as body mass (BM), height, waist circumference (WC), hip circumference (HC) and triceps, subscapularis, suprailiac, and midcalf skinfold thickness (SF). The Receiver Operating Characteristic Curve (ROC) was used to analyze the predictive capacity of anthropometric indicators in the identification of HBP.

**Results:**

Higher values of Area Under the Curve (AUC) were for the anthropometric indicators BM (0.67; 95%CI: 0.62–0.72), body mass index (BMI) (0.67; 95%CI: 0.62–0.72), and WC (0.67; 95%CI: 0.62–0.71) for males. For females, no anthropometric indicator had discriminatory power for HBP screening. The cutoff points for the anthropometric indicators with discriminatory power for HBP screening in males were BM > 64.80 Kg, BMI > 21.76 Kg/m^2^, fat percentage (FP) > 15.75, waist height to ratio (WHtR) > 0.41, WC > 73.00 cm, and HC > 92.25 cm.

**Conclusion:**

Anthropometric indicators of body adiposity had greater discriminatory power of HBP screening in males. For females, caution is suggested because the anthropometric indicators showed AUC values (95%CI) below 0.60.

## Introduction

Blood pressure is an important indicator of cardiovascular and metabolic health. Children and adolescents with high blood pressure levels are highly likely of becoming hypertensive adults. Therefore, early diagnosis and treatment can prevent long-term adverse cardiovascular events (1). Recently, a study has shown that high blood pressure (HBP) at younger ages (6 to 12 years old) may be associated with damage targeting organs such as the heart, brain, kidneys, retina, and blood vessels (2).

Epidemiological studies have shown differences between countries in the prevalence of HBP such as Africa 12.7%, China 7.7%, India 7.6%, United States 13.6%, and Brazil 14.3% (3–7). The southern region of Brazil is one of the regions with the highest prevalence of BPH in adolescents (7). Data on the prevalence of overweight in the same population is worrying, such as Mediterranean region 25.0% (2 to 13 years old), Atlantic region 19.3% (2 to 13 years old), in China, overweight is 13.2% and obesity 9.3% (adolescents aged 12–18 years), the United States, overweight prevalence is 38.7% (adolescents aged 12–15 years) and 41.5% (adolescents aged 16–19 years), and in Brazil, overweight prevalence is 17.5% and obesity is 11.7% (8–11). The southern region of Brazil has the highest prevalence of overweight adolescents in the country (18.2%) (12).

In this sense, studies have pointed out that excess weight (overweight and obesity) in the pediatric population is associated with higher blood pressure values (3, 4, 6, 13). Therefore, when body mass index (BMI) reaches the 85th percentile (overweight) in adolescents over 10 years of age, the risk of developing arterial hypertension in adulthood increases (14, 15). Furthermore, during childhood, excess weight can cause endothelial damage, consequently atherosclerosis and less arterial stiffness (16–18).

Obesity is already considered the cardiovascular risk factor with the greatest association with HBP (19). Previous studies have identified that anthropometric indicators such as BMI, waist circumference (WC), waist-to-height ratio (WHtR), waist-to-hip ratio (WHR), body adiposity index (BAI), conicity index (C), skinfolds (SF), and adiposity body shape index (ABSI) are effective in diagnosing body fat and are associated with HBP in children and adolescents (6 to 19 years old) (20–28).

However, different cutoff points have been used to classify anthropometric indicators and there is no standardization regarding HBP screening in adolescents. This is evident when verifying the different methodological procedures used such as the various protocols for assessing and measuring blood pressure, different instruments used, high range of age groups analyzed, intervals between measurements, number of incongruent measurements, and lack of consensus regarding the best cutoff point for each anthropometric indicator for HBP screening in children and adolescents (5 to 19 years old) (23, 28–36). In addition, some studies do not show diagnostic accuracy measures such as positive predictive values (PPV), negative predictive values (NPV), positive likelihood ratio (LR+), negative likelihood ratio (LR-), and area under the curve (AUC), which could provide more accurate information about cutoff points to identify the target condition or the individual's health (37).

Considering the relationship between overweight and obesity with HBP in adolescents, the use of anthropometric measures of body adiposity to identify a possible association with HBP in the pediatric population may be an effective and applicable strategy for health professionals to identify risk factors for cardiovascular diseases (13). In addition, our investigation proposes to estimate a greater number of measures of diagnostic accuracy to determine cutoff points of anthropometric indicators of body adiposity for HBP screening. This is important because the literature will have more detailed information on the cutoff points and the reader will be able to choose the anthropometric indicator that best suits the population of interest. Therefore, this study aimed to propose cutoff points for anthropometric indicators for HBP screening in adolescents and to identify, among these indicators, those more accurately for boys and girls.

## Materials and Methods

### Research Characterization

This is an observational study with a cross-sectional design using data from the school-based population research macro-project entitled Brazilian Guide for the Assessment of Physical Fitness and Health-Related Life Habits – Stage II.

### Ethical Aspects

The ethics committee in research approved this study with human beings of the Federal University of Santa Catarina, under protocol No. 3.523.470 of August 21, 2019. All adolescents who participated in the research signed the consent form and, for those aged <18 years, parents/guardians signed the Free and Informed Consent Form.

### Study Location

The study was carried out in the city of São José, located in the state of Santa Catarina, southern Brazil. The Municipal Human Development Index (MHDI) of São José was 0.809 in 2010. The percentage of young people aged 15 to 17 years with the complete elementary school was 70.94%, with a life expectancy of 77.81 years, *per capita* income of R$ 1.157,43, GINI index (income concentration of a given region) of 0.44, and low-income percentage of 1.36% (38).

### Eligible Population and Sample

The target population of this research was adolescents aged 14 to 19 years enrolled in state high schools in the city of São José, SC, Brazil. The sampling process was determined in two stages: stratified by state public high schools and cluster of classes considering study shift and grade. State schools with Youth and Adult Education (EJA) that received adolescents with some type of intellectual disability were not eligible for this study.

Based on these criteria and according to the information from the State Department of Education, the municipality had 11 eligible schools, totaling 5,411 students enrolled in the first half of 2019, and for every six students in the day shift (morning, afternoon, full-time), one student was enrolled in the night shift. In the first stage, the school density was adopted as a stratification criterion (size: small, with <200 students; medium, with 200–499 students; and large, with 500 students or more). Thus, schools that predominated according to size were randomly selected, totaling seven schools. In the second stage, study shift and grade were considered.

To determine the sample size of the macro-project that resulted in different subprojects, it was decided to calculate the sample size for prevalence studies (39). A confidence level of 1.96 (95% confidence interval), tolerable error of 3.5 percentage points, prevalence of 50% (unknown outcome), and design effect of 1.5 were adopted (39). To minimize possible losses and refusals, 20% were added (40). With these parameters, the required sample size was 1,233 students. Due to cluster sampling, all students belonging to classes were invited to participate in the research.

The sample size of day shift students was 606 and 28 students for the night shift. Regarding the grades of education, 276 students from the first year of high school, 200 students from the second year of high school, and 158 students from the third year of high school were evaluated.

### Eligibility Criteria

Adolescents who refused to participate in the study, those with a physical disability that prevented them from performing physical tests, and those who did not return the Free and Informed Consent Form signed by parents or guardians (aged <18 years) or by themselves (aged ≥ 18 years) were excluded from the research.

### Data Collection

Data collection was performed using a self-administered questionnaire composed of demographic, socioeconomic, lifestyle, and sexual maturation sections. In addition, anthropometric measurements (body mass, height, perimeters, and skinfolds) and blood pressure (systolic and diastolic blood pressure) were performed.

Undergraduate and graduate Physical Education and Nutrition students with availability to carry out fieldwork were selected. Study coordinators carried out the selection and training of the team. To minimize evaluation errors in anthropometric measurements, the intra- and inter-evaluator technical measurement error (TME) of research anthropometrists was calculated during training, having as reference measurements performed by anthropometrists with level 3 certification of the International Society for the Advancement of Kinanthropometry (ISAK) (measurements used as a reference for comparisons) (Supplementary Material 1). All TME values were considered adequate, as recommended in the literature (41).

### Dependent Variable

The dependent variable of the present study was blood pressure (systolic—SBP and diastolic—DBP), considering the average of two measurements performed on each adolescent (one at the beginning of data collection and another between 10 and 15 min after the first measurement) (42). However, if there was a difference <10 mmHg for SBP and/or DBP between the two measurements, a third measurement was performed, adopting the mean of the lowest blood pressure measurements and excluding the highest (43). The rest time before the first measurement was at least 10 min, also for the third measurement, if necessary. Blood pressure was measured on the right arm supported on a table at heart level and with palm facing up. To perform this measurement, the subject was seated, legs uncrossed, and feet on the floor (44). Electronic arm sphygmomanometers with a digital reading system (Omron® model HEM 742, Kyoto, Japan), previously and adequately validated for Brazilian adolescents (45), were used to measure blood pressure levels. Individuals were recommended not to smoke, drink coffee, and ingest black tea and alcoholic beverages, not to perform physical activities of moderate to vigorous intensity 12 h before, and to empty bladder before blood pressure measurement (44).

Blood pressure was continuously and dichotomously analyzed (HBP: yes/no). Adolescents aged 13 to 17 years with SBP values ≥ 120 and/or DBP ≥ 80 mmHg and adolescents aged 18 to 19 years with SBP values ≥ 140 and/or DBP ≥ 90 mmHg were considered to have HBP (44).

### Independent Variables

The body mass (BM), height, two perimeters, and skinfolds (SF) measurements were performed: WC and hip circumference (HC), triceps, subscapularis, suprailiac, and mid-calf SFs according to literature recommendations (46) (Supplementary Material 2), and the following anthropometric indicators were calculated: BMI, fat percentage (FP), WHtR, WC, C Index, BAI, WHR, and ABSI.

BMI was calculated through BM and height measurements. FP was calculated from height and WC measurement through the equation: FP = 64–[20 x (Height (m)/WC (m)] + (12 x Sex), with zero (0) for males and one (1) for females (47).

Using WC, HC, and height measurements, WHtR and WHR indicators were calculated. The conicity index (C index) was calculated through WC, BM, and height measurements: C index = WC (m) / 0.109 x [√BM (Kg) / height (m)] (48). In addition, from HC and height values, the body adiposity index (BAI) was calculated (49). ABSI was calculated through WC, BMI, and height measurements using the equation: ABSI = WC (m) / (^3^√(BMI^2^) x √height) (22). Furthermore, SF was continuously analyzed by the sum of SFs.

### Sample Characterization Variables

The characterization variables of this study were: sociodemographic indicators (economic level and skin color), physical activity, eating habits, cigarette use, sleep quality, and sexual maturation, collected through a self-administered questionnaire.

The economic level was estimated using a questionnaire from the Brazilian Association of Research Companies (50). This questionnaire estimates the purchasing power of households. This questionnaire estimates the purchasing power of families based on different items that are present in adolescents' homes (bathroom, automobiles, microcomputer, dishwasher, refrigerator, freezer, washing machine, DVD, microwave, motorcycle, and clothes dryer). From the answers, the economic level can be categorized as decreasing level of purchasing power (from A to E). In the present study, the economic level was dichotomized into “high” (level A and B) and “low” (level C, D, and E). Skin color was assessed by the Brazilian census methodology that uses the words white, brown, black, yellow and indigenous to classify people's skin color or race. The following classification was adopted: “white” and “brown, yellow, indigenous and black”.

Physical activity was assessed using the question “During the last 7 days, on how many days were you physically active for at least 60 minutes a day?” from the Youth Risk Behavior Surveillance System (YRBSS) questionnaire used in the United States, translated and validated for Brazil (51). The questionnaire had a Kappa agreement index for the Brazilian population of 68.6% and the question used in the present study had a Kappa agreement index of 37.2% (51). Responses were categorized as “physically active” when active for 7 days and “not physically active” when active <7 days a week (52).

Eating habits were assessed using the question “Do you eat a balanced diet?” of the Fantastic Lifestyle questionnaire (53), translated and validated for the Brazilian population (54). The questionnaire had a Kappa agreement index for the Brazilian population of 68.6% and the question used in the present study had a Kappa agreement index of 72% (54). The instrument's response options were categorized into “inadequate” (option 0 – almost never; option 1 – rarely; and option 2 – sometimes), and “adequate” (option 3 – relatively often; and option 4 – almost always), as explained in the questionnaire itself (55).

Cigarette use was assessed using the question “Do you smoke cigarettes?” from the Fantastic Lifestyle questionnaire (53), with the question showing 86% of the Kappa agreement index in the Brazilian population (54). The instrument's response options were categorized into: “currently smoke” (option 0 – more than 10 per day; and option 1 – 1 to 10 a day) and “do not currently smoke” (option 2 – none in the last 6 months; option 3 – none in the past year; and option 4 – never smoked).

Sleep quality was assessed using the question “Do you sleep well and do you feel rested?” from the Fantastic Lifestyle questionnaire (53). This question had a Kappa agreement index of 55% for the Brazilian population (54). The instrument's response options were categorized into “adequate” (option 3 - relatively often; and option 4 - almost always) and “inadequate” (option 0 – almost never; option 1 – rarely; and option 2 – sometimes), according to study that evaluated the same variable (56).

For the self-assessment of sexual maturation, we used Tanner's scales (57), validated and reproduced for the Brazilian population with an agreement of 60.9 to 71.3% (58). Sexual maturation stages were indicated by self-assessment (figures) based on pubic hair development (males and females). Stage 1 represents the pre-pubertal stage, stages 2, 3, and 4 represent puberty, and stage 5 the post-pubertal stage. Adolescents were classified as pre-pubertal, pubertal, and post-pubertal, similarly to another study (59).

### Statistical Analysis

Initially, data were entered with a double entry in the Epi Data 3.0 software. From there, descriptive statistics (mean, standard deviation, and frequencies) were performed. Differences between sexes and ages were analyzed using the Student's *t*-test for independent samples. Data normality was verified using the Shapiro–Wilk test and, if data did not present normal distribution, the non-parametric Mann–Whitney's test was performed.

The chi-square test was used to verify differences in prevalence between sexes and the physical, sociodemographic, and lifestyle characteristics of male and female adolescents. The ROC curve (Receiver Operating Characteristic Curve) was used to analyze the predictive capacity of anthropometric indicators to identify high SBP and DBP and to find the best cutoff points that identify this association (60). For the present study, the cutoff points for anthropometric indicators of obesity were those with sensitivity above 70% to avoid the maximum number of false-negative results (61).

AUC >0.6 was considered sufficient, regardless of *p*-value, and AUC >0.7 was considered to have good diagnostic accuracy (37). In addition, sensitivity, specificity, PPV, NPV, LR+, and LR- values were calculated for all cutoff points of anthropometric indicators to identify high SBP and DBP values to interpret such cutoff points. Analyses were performed stratified by sex (male and female) and the significance level was set at 5%. Analyses were performed using the MedCalc 19.5.3 statistical software and Statistical Package for the Social Sciences software (SPSS Statistics, Chicago, USA), version 17.0.

## Results

A total of 634 adolescents aged 14 to 19 years completed all assessments of the present study. Most adolescents were male (62.5%). Significant differences were observed between sexes, with higher BM, height, WHR, ABSI, C Index, WC, and SBP values for male adolescents and higher FP, BAI, triceps, subscapularis, suprailiac, calf SFs, and sum of SFs and DBP values for female adolescents (Table 1).

**Table 1:** General characterization of the sample (*n* = 634) adolescents from São José, SC, Brazil, 2019.

**Variables**	**Male (*n* = 396, 62.5%)**	**Female (*n*= 238, 37.5%)**	***p*-value**
	**Mean (SD)**	**Mean (SD)**	
Age (years old)	16.63 (1.02)	16.62 (1.06)	0.92
BM (Kg)	68.11 (13.52)	58.63 (12.31)	**<0.01**
Height (cm)	173.62 (7.16)	160.55 (5.82)	**<0.01**
BMI (Kg/m^2^)	22.58 (4.23)	22.72 (4.58)	0.68
FP	17.30 (4.91)	29.41 (5.86)	**<0.01**
WHtR	0.43 (0.05)	0.44 (0.06)	0.46
WHR	0.78 (0.04)	0.72 (0.06)	**<0.01**
BAI	24.10 (4.04)	29.50 (4.65)	**<0.01**
ABSI	0.072 (0.003)	0.069 (0.004)	**<0.01**
C Index	1.10 (0.04)	1.07 (0.06)	**<0.01**
WC (cm)	75.17 (8.60)	70.09 (9.85)	**<0.01**
HC (cm)	96.15 (8.73)	96.49 (8.96)	0.64
Triceps skinfold (mm)	11.18 (5.16)	17.88 (5.72)	**<0.01**
Subescapularis skinfold (mm)	10.72 (5.02)	14.55 (6.92)	**<0.01**
Supra iliac skinfold (mm)	13.91 (7.30)	17.50 (6.74)	**<0.01**
Calf skinfold (mm)	9.91 (4.85)	17.17 (6.86)	**<0.01**
Sum of skinfolds (mm)	45.73 (20.49)	67.10 (23.19)	**<0.01**
SBP (mmHg)	109.78 (21.71)	102.76 (15.62)	**<0.01**
DBP (mmHg)	65.21 (8.08)	67.35 (8.71)	**<0.01**

*n, sample size; %, percentage; t-test for independent samples; Mann–Whitney's non-parametric test; SD, standard deviation; BM, Body mass; BMI, Body mass index; FP, fat percentage; WHtR, waist-to-height ratio; WHR, waist-hip ratio; BAI, body adiposity index; ABSI, adiposity body shape index; C-index, conicity index; WC, waist circumference; HC, hip circumference; SBP, systolic blood pressure; DBP, diastolic blood pressure; the p-value in bold means a statistically significant difference (p < 0.05) between males and females*.

Yellow/brown/indigenous/black skin color students were more frequent in males (48.2%), when compared to female adolescents (38.6%). Male adolescents had higher prevalence of HBP (29.8%) when compared to female adolescents (11.3%) (*p*-value = 0.00) (Table 2).

**Table 2:** Physical, sociodemographic, and lifestyle characteristics of public school students in São José, SC, Brazil.

		**General** ***(n*** **=** **634)**	
		**Male *n* = 396 (%)**	**Female *n* = 238 (%)**	
**Variables**	***n* = 634**			***p*-value**
**Maturation**	*n* = 598			0.33
Pre-pubertal		40 (10.7)	16 (7.2)	
Pubertal		250 (66.7)	158 (70.9)	
Post-pubertal		85 (22.7)	49 (22.0)	
**Economic level**	*n* = 634			0.38
High		260 (65.7)	148 (62.2)	
Low		136 (34.3)	90 (37.8)	
**Skin color**	*n* = 619			**<0.05**
White		200 (51.8)	143 (61.4)	
Brown/yellow/indigenous/black		186 (48.2)	90 (38.6)	
**Physical activity level**	*n* = 618			0.48
Physically active		23 (6.0)	11 (4.7)	
Not physically active		360 (94.0)	224 (95.3)	
**Balanced diet**	*n* = 634			0.42
Adequate		68 (17.2)	35 (14.7)	
Inadequate		328 (82.8)	203 (85.3)	
**Smoke cigarettes**	*n* = 616			0.45
Not currently smoker		370 (96.1)	219 (94.8)	
Currently smoker		15 (3.9)	12 (5.2)	
**Sleep quality**	*n* = 620			0.34
Adequate		147 (37.9)	79 (34.1)	
Inadequate		241 (62.1)	153 (65.9)	
**HBP**	*n* = 634			
No		278 (70.2)	211 (88.7)	**<0.01**
Yes		118 (29.8)	27 (11.3)	

*n, sample size (number of students with information about the variable); %, percentage; Chi-square test; HBP, High blood pressure; the p-value in bold means a statistically significant difference (p < 0.05) in the skin color and HBP between males and females*.

For male adolescents, anthropometric indicators BM, BMI, FP, WHtR, WC, and HC showed sufficient AUC (95%CI of AUC > 0.60), ranging from 0.65 to 0.67, with 95%CI ranging from 0.60 to 0.72. Likewise, LR+ values were >1, demonstrating that individuals are more likely of having the disease with a positive result, and with LR- values lower than 50, demonstrating that an individual with a negative result is 50% less likely of having the disease, for example, an adolescent with BM >64.8 kg is 1.73 times more likely of having HBP than an adolescent with BM <64.8 kg and an adolescent with BM <64.8 kg is 47% more likely of having HBP (Table 3).

**Table 3:** Area under the curve, cutoff point, sensitivity values, specificity values, positive likelihood ratio, negative likelihood ratio, positive predictive values, and negative predictive values of anthropometric indicators for high blood pressure screening in male adolescents (*n* = 396).

**Variable**	**AUC (95%CI)**	***p*-value**	**Cut off point**	**Sensitivity**	**Specificity**	**LR+**	**LR–**	**PPV**	**NPV**
BM (kg)	0.67 (0.62–0.72)	0.00	>64.80	72.88 (63.90–80.70)	57.91 (51.90–63.80)	1.73 (1.50–2.10)	0.47 (0.30–0.60)	42.40 (38.10–46.70)	83.40 (78.60–87.30)
Height (cm)	0.54 (0.49–0.59)	0.21	>169.50	79.66 (71.30–86.50)	29.14 (23.90–34.90)	1.12 (1.00–1.30)	0.70 (0.50–1.00)	32.30 (29.80–34.90)	77.10 (69.30–83.40)
BMI (kg/m^2^)	0.67 (0.62–0.72)	0.00	>21.76	70.34 (61.20–78.40)	59.71 (53.70–65.50)	1.75 (1.50–2.10)	0.50 (0.40–0.70)	42.60 (38.10–47.10)	82.60 (77.90–86.40)
FP	0.65 (0.60–0.70)	0.00	>15.75	78.81 (70.30–85.80)	48.56 (42.50–54.60)	1.53 (1.30–1.80)	0.44 (0.30–0.60)	39.40 (35.90–43.00)	84.40 (78.90–88.60)
WHtR	0.65 (0.60–0.70)	0.00	>0.41	78.81 (70.30–85.80)	48.56 (42.50–54.60)	1.53 (1.30–1.80)	0.44 (0.30–0.60)	39.40 (35.90–43.00)	84.40 (78.90–88.60)
WHR	0.57 (0.52–0.61)	0.03	>0.75	85.59 (77.90–91.40)	28.42 (23.20–34.10)	1.20 (1.10–1.30)	0.51 (0.30–0.80)	33.70 (31.40–36.00)	82.30 (74.20–88.20)
BAI	0.62 (0.57–0.67)	0.00	>22.53	70.34 (61.20–78.40)	44.60 (38.70–50.70)	1.27 (1.10–1.50)	0.66 (0.50–0.90)	35.00 (31.50–38.70)	78.00 (72.30–82.80)
ABSI	0.57 (0.52–0.62)	0.02	<0.073	70.34 (61.20–78.40)	38.49 (32.70–44.50)	1.14 (1.00–1.30)	0.77 (0.60–1.11)	32.70 (29.50–36.10)	75.40 (69.00–80.70)
C index	0.56 (0.51–0.61)	0.05	>1.09	70.34 (61.20–78.40)	38.85 (33.10–44.90)	1.15 (1.00–1.30)	0.76 (0.60–1.00)	32.80 (29.60–36.20)	75.50 (69.30–80.90)
WC (cm)	0.67 (0.62–0.71)	0.00	>73.00	72.88 (63.90–80.70)	57.91 (51.90–63.80)	1.73 (1.50–2.10)	0.47 (0.30–0.60)	42.40 (38.10–46.70)	83.40 (78.60–87.30)
HC (cm)	0.66 (0.61–0.71)	0.00	>92.25	81.36 (73.10–87.90)	47.84 (41.80–53.90)	1.56 (1.40–1.80)	0.39 (0.30–0.60)	39.80 (36.50–43.30)	85.80 (80.30–90.00)
Triceps skinfold (mm)	0.61 (0.56–0.66)	0.00	>8.90	73.73 (64.80–81.40)	45.68 (39.70–51.70)	1.36 (1.20–1.60)	0.58 (0.40–0.80)	36.60 (33.10–40.20)	80.40 (74.70–85.00)
Subescapularis skinfold (mm)	0.62 (0.57–0.67)	0.00	>8.40	72.88 (63.90–80.70)	42.45 (36.60–48.50)	1.27 (1.10–1.50)	0.64 (0.50–0.90)	35.00 (31.60–38.40)	78.70 (72.70–83.60)
Supra iliac skinfold (mm)	0.61 (0.56–0.66)	0.00	>9.80	72.88 (63.90–80.70)	42.81 (36.90–48.90)	1.27 (1.10–1.50)	0.63 (0.50–0.90)	35.10 (31.80–38.60)	78.80 (72.90–83.70)
Calf skinfold (mm)	0.60 (0.55–0.65)	0.00	>7.95	70.34 (61.20–78.40)	44.24 (38.30–50.30)	1.26 (1.10–1.50)	0.67 (0.50–0.90)	34.10 (30.70–37.70)	78.40 (72.80–83.20)
Sum of skinfolds (mm)	0.62 (0.57–0.67)	0.00	>36.00	70.34 (61.20–78.40)	45.32 (39.40–51.40)	1.29 (1.10–1.50)	0.65 (0.50–0.90)	35.30 (31.80–39.00)	78.30 (72.60–83.00)

*95%CI, confidence interval at 95%; AUC, area under the curve (with lower bound CI95% > 0.60); LR+, Positive likelihood ratio; LR–, Negative likelihood ratio; PPV, Positive predictive values; NPV, Negative predictive values; BM, Body mass; BMI, Body mass index; FP, fat percentage; WHtR, waist–to–height ratio; WHR, Waist–to–hip ratio; BAI, Body adiposity index; ABSI, body shape index; C Index, conicity index; WC, waist circumference; HC, hip circumference*.

Table 3 presents PPV and NPV, with an emphasis on NPV, demonstrating that the use of cutoff points of anthropometric indicators mentioned above presents an 80% chance of not having the disease when the test is negative, that is, if BM <64.8 kg, the adolescent is 83% more likely of not having HBP (Table 3). Anthropometric indicators such as BAI, triceps skinfold, subscapularis skinfold, supra iliac skinfold, calf skinfold, and sum of skinfolds, despite having AUC >0.60, showed 95%CI <0.60. In addition, these anthropometric indicators showed LR- ≤ 50%, demonstrating that an individual, even with a negative result, has a more than 50% chance of having the disease (Table 3).

Regarding cutoff points of anthropometric indicators with better accuracy for HBP screening, BM had cutoff point of >64.8 kg, BMI >21.76 kg/m^2^, FP >15.75%, WHtR >0.41, WC >73.00 cm, and HC >92.25 cm. Furthermore, both anthropometric indicators showed sensitivity >70%, indicating that for every 100 adolescents evaluated, 70 have HBP. However, the same cannot be said about specificity, which presented values <60%, indicating that for every 100 adolescents evaluated, <60 are diagnosed as healthy, and the others are diagnosed with HBP (Table 3).

For female adolescents, BM and height presented AUC >0.60, however, with 95%CI <0.60. The cutoff points for anthropometric indicators with the best accuracy to identify HBP in female adolescents were BM >54.10 kg and height >158.50 cm (Table 4). Both anthropometric indicators showed sensitivity >70%, demonstrating that for every 100 adolescents evaluated, 70 have HBP; however, with specificity <43%, indicating that for every 100 adolescents evaluated, only 43 are diagnosed healthy, and the other 57 have HBP even though they are below the cutoff point (Table 4).

**Table 4:** Area under the curve, cutoff point, sensitivity values, specificity values, positive likelihood ratio, negative likelihood ratio, positive predictive values, and negative predictive values of anthropometric indicators for high blood pressure screening in female adolescents (*n* = 238).

**Variable**	**AUC (95%CI)**	***p*–value**	**Cut off point**	**Sensitivity**	**Specificity**	**LR+**	**LR–**	**PPV**	**NPV**
BM (kg)	0.62 (0.55–0.68)	0.07	>54.10	70.37 (49.80–86.20)	43.13 (36.30–50.10)	1.24 (0.90–1.60)	0.69 (0.40–1.30)	13.60 (10.70–17.10)	92.00 (86.20–95.40)
Height (cm)	0.64 (0.58–0.70)	0.01	>15.50	88.89 (70.80–97.60)	42.18 (35.40–49.20)	1.54 (1.30–1.80)	0.26 (0.09–0.80)	16.40 (14.10–18.90)	96.80 (91.00–98.90)
BMI (kg/m^2^)	0.57 (0.50–0.63)	0.28	>20.13	70.37 (49.80–86.20)	34.60 (28.20–41.40)	1.08 (0.80–1.40)	0.86 (0.50–1.60)	12.10 (9.50–15.10)	90.20 (83.30–94.40)
FP	0.53 (0.46–0.59)	0.69	>24.75	70.37 (49.80–86.20)	23.70 (18.10–30.00)	0.92 (0.70–1.20)	1.25 (0.70–2.30)	10.50 (8.30–13.20)	86.30 (77.00–92.20)
WHtR	0.53 (0.46–0.59)	0.69	>0.39	70.37 (49.80–86.20)	23.70 (18.10–30.00)	0.92 (0.70–1.20)	1.25 (0.70–2.30)	10.50 (8.30–13.20)	86.30 (77.00–92.20)
WHR	0.52 (0.45–0.58)	0.79	>0.69	70.37 (49.80–86.20)	22.75 (17.30–29.00)	0.91 (0.70–1.20)	1.30 (0.70–2.50)	10.40 (8.20–13.00)	85.80 (76.20–91.90)
BAI	0.50 (0.44–0.57)	0.98	>26.43	70.37 (49.80–86.20)	27.96 (22.00–34.50)	0.98 (0.80–1.30)	1.06 (0.60–2.00)	11.10 (8.80–13.90)	88.10 (79.90–93.20)
ABSI	0.51 (0.44–0.57)	0.94	>0.067	70.37 (49.80–86.20)	27.96 (22.00–34.50)	0.98 (0.80–1.30)	1.06 (0.60–2.00)	11.10 (8.80–13.90)	88.10 (79.90–93.20)
C index	0.53 (0.46–0.59)	0.67	>1.03	70.37 (49.80–86.20)	25.12 (19.40–31.50)	0.94 (0.70–1.20)	1.18 (0.60–2.20)	10.70 (8.50–13.40)	86.90 (78.10–92.60)
WC (cm)	0.57 (0.50–0.63)	0.32	>64.20	70.37 (49.80–86.20)	29.86 (23.80–36.50)	1.00 (0.80–1.30)	0.99 (0.50–1.80)	11.30 (9.00–14.20)	88.80 (81.00–93.60)
HC (cm)	0.58 (0.51–0.64)	0.22	>93.25	70.37 (49.80–86.20)	43.13 (36.30–50.10)	1.24 (0.90–1.60)	0.69 (0.40–1.30)	13.60 (10.70–17.10)	92.00 (86.20–95.40)
Triceps skinfold (mm)	0.54 (0.48–0.61)	0.51	>15.40	70.37 (49.80–86.20)	39.34 (32.70–46.30)	1.16 (0.90–1.50)	0.75 (0.40–1.40)	12.90 (10.20–16.20)	91.20 (85.10–95.00)
Subescapularis skinfold (mm)	0.50 (0.44–0.57)	0.98	>9.80	70.37 (49.80–86.20)	27.96 (22.00–34.50)	0.98 (0.80–1.30)	1.06 (0.60–2.00)	11.10 (8.80–13.90)	88.10 (79.90–93.20)
Supra iliac skinfold (mm)	0.53 (0.46–0.59)	0.68	≤ 23.00	70.37 (49.80–86.20)	19.43 (14.30–25.40)	0.87 (0.70–1.10)	1.52 (0.80–2.90)	10.00 (7.90–12.50)	83.70 (73.00–90.70)
Calf skinfold (mm)	0.57 (0.50–0.63)	0.33	>13.77	70.37 (49.80–86.20)	39.34 (32.70–46.30)	1.16 (0.90–1.50)	0.75 (0.40–1.40)	12.90 (10.20–16.20)	91.20 (85.10–95.00)
Sum of skinfolds (mm)	0.52 (0.46–0.59)	0.74	>52.20	70.37 (49.80–86.20)	29.86 (23.80–36.50)	1.00 (0.80–1.30)	0.99 (0.50–1.80)	11.30 (9.00–14.20)	88.80 (81.00–93.60)

*95%CI, confidence interval at 95%; AUC, area under the curve (with lower bound 95%CI ≥ 0.60); LR+, Positive likelihood ratio; LR–, Negative likelihood ratio; PPV, Positive predictive values; NPV, Negative predictive values; BM, Body mass; BMI, Body mass index; FP, fat percentage; WHtR, waist–to–height ratio; WHR, Waist–to–hip ratio; BAI, Body adiposity index; ABSI, body shape index; C-Index, conicity index; WC, waist circumference; HC, hip circumference*.

In addition, LR+ values were >1, demonstrating that individuals are more likely of having the disease with the positive result; however, 95%CI presented by BM was between 0 and 1, indicating that the chances of disease are equal between individuals with and without the disease and with LR- value ≥50, demonstrating that an individual with a negative result is 50% more likely of having the disease. Unlike height, which presented LR+ >1 and LR– values close to zero, demonstrating that individuals with the negative result are less likely of having the disease, for example, an adolescent with a height >158.50 cm is 1.54 times more likely of having HBP that an adolescent with height <158.50 cm and, in the same way, an individual with height <158.50 cm with 26% chance of having HBP. Both anthropometric indicators showed high NPV, demonstrating that the use of cutoff points showed a probability of more than 90% of not having the disease when the test is negative, that is, an adolescent with a height <158.50 cm is 90% more likely of not having HBP (Table 4).

The other anthropometric indicators (BMI, FP, WHtR, WHR, BAI, ABSI, C index, WC, HC, triceps skinfold, subscapularis skinfold, supra iliac skinfold, calf skinfold, and sum of skinfolds), which did not show sufficient AUC (95%CI AUC <0.60), also showed LR+ values close to 1 or below 1, demonstrating that individuals who presented a positive test result would have the same chances of having the disease when compared to those who presented a negative result (Table 4).

## Discussion

In the present study, the predictive capacity and proposition of cutoff points of anthropometric indicators to identify HBP in male and female adolescents were investigated. For males, indicators BM, BMI, FP, WHtR, WC, and HC presented sufficient AUC (i.e., 95%CI AUC > 0.60). For females, no anthropometric indicator had discriminatory power for HBP screening. This study is not intended to replace the clinical diagnosis, but to allow the identification, in a simple way, of adolescents who are more likely of having HBP in the initial screening and who would need further care and follow-up.

High values in anthropometric indicators of body adiposity are associated with HBP in adolescents (1, 2, 13, 21–23), and this association is justified by the high concentration of fatty acids in subjects with high body fat. This condition (i.e., high concentration of fatty acids) causes insulin resistance and may induce the renal system to retain more sodium. This retention increases activation of the sympathetic nervous system, which results in increased activity of the renin-angiotensin system and increases blood pressure (62).

This study proposed to update cutoff points of anthropometric indicators for HBP screening in Brazilian adolescents and added more anthropometric indicators for the debate with literature, such as FP, ABSI, triceps, subscapular, suprailiac, calf SFs, and sum of SFs, which were not present in studies published with Brazilian adolescents, in which the use of BMI, WHtR, and WC prevailed (21, 23, 63, 64). Furthermore, previous Brazilian studies have shown sensitivity and specificity values (21, 23, 63, 64), and in two studies (23, 63), the 95%CI sensitivity of anthropometric indicators was below 0.60, and in one study (23), LR+ and LR– values were not presented as 95%CI. AUC allows evaluating the performance on the test, in this case, the performance of anthropometric indicators for HBP screening in adolescents. If the AUC is less than 0.60, the accuracy of the diagnostic test is considered poor, random (37), and may or may not identify HBP in adolescents.

In the present study, for males, anthropometric indicators BM, BMI, FP, WHtR, WC, and HC presented AUC (95%CI >0.60), demonstrating sufficient accuracy for HBP screening (37). The BMI cutoff point for HBP screening in males in the present study was >21.76 kg/m^2^, which is lower than cutoff points in other studies (21, 23, 65, 66). If compared to the BMI cutoff proposed by the World Health Organization (67) to identify obesity in adolescents [14 years (21.8 kg/m^2^), 15 years (22.7 kg/m^2^), 16 years (23.5 kg/m^2^), 17 years (24.3 kg/m^2^), 18 years (24.9 kg/m^2^), and 19 years (24.9 kg/m^2^)], the cutoff point of the present study was lower, which may have occurred due to the following factors: data from the World Health Organization are longitudinal population-based with the participation of several countries and stratified by age, while the present study has a cross-sectional design, carried out in a certain geographic region of Brazil, without stratification by age, and using the average age of adolescents. Although the cutoff point is considered low compared to other studies, this was one of the indicators that showed good accuracy for HBP screening in male adolescents in the present study, since it had an LR+ value close to two, demonstrating that an individual with a positive result is more likely of having the disease, LR– of 0.50, that is, an individual with a negative result is 50% more likely of having the disease and NPV of 82.50 (95%CI: 77.80–86.40).

Regarding the cutoff point for WHtR (>0.41) identified in this study for HBP screening in males, another study presented the same cutoff point of 0.41 (68). The cutoff point of 0.41 for WHtR is below cutoff points in other studies (21, 23, 63, 65, 66, 69–71). Since 1995 (72), the year of the creation of anthropometric indicator WHtR, a universal cutoff point (0.50) has been established to identify overweight individuals from those with normal weight and to be considered a risk factor for cardiovascular diseases, among them, hypertension, which can be used for both sexes and different age and ethnic groups (72, 73). However, this cutoff point of 0.50 was not created from AUC, therefore without diagnostic tests. Furthermore, WC measurement was performed on the umbilical line and not on the smallest portion of the trunk, which could therefore generate differences with the standardization used in the present study (72). In addition, different protocols for measuring WC were used by studies (21, 65, 66), which may interfere with the WHtR results and, consequently, with cutoff point values proposed by these studies.

The present study also identified other anthropometric measures and indicators with good accuracy for HBP screening in male adolescents, namely BM, FP, and HC. BM in the present study presented a sensitivity value of 72.88 (95%CI: 63.90–80.70), LR+ close to two, LR– <0.50, and NPV of 83.40 (95%CI: 78.60–87.30), demonstrating good accuracy for HBP screening in male adolescents, with a cutoff point of >64.80 kg. The other studies in the literature only used BM measurement to calculate other anthropometric indicators. In this sense, this study presents this anthropometric measurement, which is easy to be performed by any professional, as a strategy to be used for the initial HBP screening.

In the present study, HC was analyzed in isolation and showed good accuracy for HBP screening in male adolescents, when high, with a cutoff point of >92.25 cm. In addition, it was used in conjunction with WC and height to calculate other anthropometric indicators, such as WHR and BAI, but without showing good AUC accuracy to identify HBP in male and female adolescents. In other studies, HC was not analyzed in isolation, only in conjunction with other anthropometric measures to calculate BAI (23) and WHR (69) indicators. The study that used BAI showed good accuracy for HBP screening in male and female adolescents and the study that used WHR, carried out only with female adolescents, showed good accuracy for HBP screening, and both studies adopted AUC (95%CI >0.50), different from the present study that adopted AUC (95%CI >0.60) (23, 69).

In the present study, FP, calculated using two anthropometric measurements (height and WC), presented an AUC of 0.65 (95%CI: 0.60–0.70) and a cutoff point of >15.75%. A study carried out with Turkish adolescents aged 11 to 17 years also verified the accuracy of FP for HBP screening in adolescents; however, it was not clear how FP was calculated and, unlike the present study, AUC >0.60 was not identified for this indicator (65). FP calculated by height and HC has not yet been validated for Brazilian adolescents (74); however, this index showed better agreement with dual-energy x-ray absorptiometry (DXA) of 0.83 and 0.86 for female and male adolescents, respectively, compared to BMI and tri-ponderal mass index (TMI), which use is recommended in health care services and the school environment (47). In addition, it presented 3.45 (95%CI: 3.26–3.63) and 3.35 (95%CI: 3.22–3.49) as mean square errors for female and male adolescents aged 15 to 19 years, respectively (47). Furthermore, FP calculated through skinfolds (triceps and subscapularis) (75) is valid for up to the age of 18 years and still depends on constants for ethnicity, which is unnecessary for the calculation of FP with the equation used in the present study that differentiates only between the sex of adolescents and presents a direct estimate of FP (47).

For female adolescents, BM and height presented AUC >0.60; however, with 95%CI <0.60, demonstrating low accuracy to identify HBP in this population. In other studies (21, 23, 63, 64, 69, 70, 76), AUC >0.60 was identified for some anthropometric indicators, such as BMI, HC, WHtR, BAI, and C Index, demonstrating good accuracy for HBP screening in female adolescents. Of these, two studies presented more diagnostic accuracy measures (LR+ and LR–) (23), PPV and NPV (69). The present study did not present anthropometric indicators with good accuracy to identify HBP in female adolescents, which can be explained by the low prevalence of HBP (11.3%), which was below other studies carried out with Brazilian adolescents (7, 77).

A debate in the literature that deserves to be highlighted is the recommended AUC values for diagnostic tests and/or screening. As the present study aimed to propose cutoff points for anthropometric indicators to perform HBP screening among Brazilian adolescents, we chose to use a reference for classifying AUC values >0.60 as sufficient to identify the disease risk (37). There are other references for satisfactory AUC values, such as > 0.70 (78, 79). As there are still debates in the literature with no conclusions on which recommendations to use, it was decided to use AUC values >0.60 aiming at an early screening of the risk of HBP.

This study had strengths and limitations. As a strong point, the fact that 16 anthropometric indicators were investigated and calculated through eight anthropometric measurements is highlighted, enabling verifying the accuracy of the HBP screening in adolescents aged 14 to 19 years. In addition, this study presented measures of diagnostic accuracy not present in other studies, providing additional information to the literature. Finally, this research calculated the technical error of measurement among anthropometrists and used a blood pressure monitor validated for adolescents (80), which demonstrates care during data collection and analysis.

As limitation, the size of the sample collected was smaller than the calculated one, which limits inferences of the study. In addition, despite the measurement of sexual maturation, it was only used to characterize adolescents and did not enter as adjustments in anthropometric indicators. Another limitation of this research was the cross-sectional design, making it impossible to establish a cause-and-effect relationship.

## Conclusion

Based on the results, it could be concluded that anthropometric indicators BM, BMI, FP, WHtR, WC, and HC have good accuracy for HBP screening in male adolescents. However, for female adolescents, anthropometric indicators did not present good diagnostic characteristics for HBP screening. Thus, it is suggested that, in a school environment, the use of anthropometric measurements is a simple, low-cost, and easy-to-apply method, which can be performed by the Physical Education teacher, being an effective strategy to contribute to obtaining information about adolescents' health.

This study is not intended to replace the clinical diagnosis of HBP through cutoff points found, but to demonstrate that male individuals with high BM, BMI, FP, WHtR, WC, and HC values should be referred for further clinical tests to verify the presence or absence of the disease.

## Data Availability Statement

The original contributions presented in the study are included in the article/Supplementary Material, further inquiries can be directed to the corresponding author/s.

## Ethics Statement

The studies involving human participants were reviewed and approved by Ethics and Research Committee, Federal University of Santa Catarina. Written informed consent to participate in this study was provided by the participants' legal guardian/next of kin.

## Author Contributions

DS contributed to the study construction, data interpretation, writing, manuscript supervision and review, and obtained approval from the Research Ethics Committee of the Institution to carry out the study. AG, LM, and AU contributed to data interpretation and manuscript editing. LB contributed to data analysis and interpretation, writing, manuscript editing and review, and acquired the authors' authorization for manuscript submission. All authors contributed to the article and approved the submitted version.

## Funding

This research was financed by the Coordenação de Aperfeiçoamento de Pessoal de Nível Superior - CAPES/PROEX (Brazil) - n° 23038.007266 - by the Graduate Program in Physical Education/UFSC.

## Conflict of Interest

The authors declare that the research was conducted in the absence of any commercial or financial relationships that could be construed as a potential conflict of interest.

## Publisher's Note

All claims expressed in this article are solely those of the authors and do not necessarily represent those of their affiliated organizations, or those of the publisher, the editors and the reviewers. Any product that may be evaluated in this article, or claim that may be made by its manufacturer, is not guaranteed or endorsed by the publisher.

## References

[1] Pinheiro G Mello J Gaya A Gaya AR. Pressão Arterial de Crianças: Associação a Indicadores Antropométricos, Composição Corporal, Aptidão Cardiorrespiratória e Atividade Física. Arq Bras Cardiol. (2021) 116:950–6. 10.36660/abc.20190520 34008820 PMC8121480

[2] Goelzer MNAP Scala LCN. Prevalência de hipertensão arterial, pré-hipertensão e fatores associados em crianças e adolescentes de escolas municipais de Cuiabá, Mato Grosso. Connection Line-Revista Eletrônica do Univag. (2020) 23:4–23. 10.18312/connectionline.v0i23.1586

[3] Noubiap JJ Essouma M Bigna JJ Jingi AM Aminde LN Nansseu JR. Prevalence of elevated blood pressure in children and adolescents in Africa: a systematic review and meta-analysis. Lancet Public Health. (2017) 2:e375–86. 10.1016/S2468-2667(17)30123-8 29253478

[4] Wang L Song L Liu B Zhang L Wu M Cao Z. Trends and status of the prevalence of elevated blood pressure in children and adolescents in China: a systematic review and meta-analysis. Curr Hypertens Rep. (2019) 21:1–12. 10.1007/s11906-019-0992-1 31599364

[5] Daniel RA Haldar P Prasad M Kant S Krishnan A Gupta SK. Prevalence of hypertension among adolescents (10-19 years) in India: a systematic review and meta-analysis of cross-sectional studies. PLoS ONE. (2020) 15:e0239929. 10.1371/journal.pone.0239929 33022021 PMC7537899

[6] Xi B Zhang T Zhang M Liu F Zong X Zhao M. Trends in elevated blood pressure among US children and adolescents: 1999–2012. Am J Hypertens. (2016) 29:217–25. 10.1093/ajh/hpv091 26158854 PMC4900148

[7] Santos JCS. Prevalência de hipertensão em crianças e adolescentes escolares do Brasil: um estudo de revisão. (Trabalho de Conclusão de Curso - Licenciatura em Educação Física) – Universidade Federal Rural de Pernambuco, Pernambuco, Brasil. (2019). Available online at: https://repository.ufrpe.br/handle/123456789/1800 (accessed June 23, 2022).

[8] Garrido-Miguel M Cavero-Redondo I Álvarez-Bueno C Rodríguez-Artalejo F Moreno LA Ruiz JR. Prevalence and trends of overweight and obesity in European children from 1999 to 2016: a systematic review and meta-analysis. JAMA Pediatr. (2019) 173:e192430–e192430. 10.1001/jamapediatrics.2019.2430 31381031 PMC6686782

[9] Zhang J Wang H Wang Z Du W Su C Zhang J. Prevalence and stabilizing trends in overweight and obesity among children and adolescents in China, 2011–2015. BMC Public Health. (2018) 18:1–7. 10.1186/s12889-018-5483-9 29716560 PMC5930802

[10] Skinner AC Ravanbakht BA Skelton JA Perrin EM Armstrong SC. Prevalence of obesity and severe obesity in US children, 1999–2016. Pediatrics. (2018) 141:3. 10.1542/peds.2017-3459 29483202 PMC6109602

[11] Pelegrini A Bim MA Souza FU Kilim KSS Pinto AA. Prevalência de sobrepeso e obesidade em crianças e adolescentes brasileiros: uma revisão sistemática. Rev Bras de Cineantropometria e Desempenho Hum. (2021) 23:e80352. 10.1590/1980-0037.2021v23e80352

[12] Simões CF Lopes WA Remor JM Locateli JC Lima FB Santos TLCD. Prevalência de excesso de peso em crianças e adolescentes brasileiros: uma revisão sistemática. Rev Bras de Cineantropometria e Desempenho Hum. (2018) 20:517–31. 10.5007/1980-0037.2018v20n4p517

[13] Quadros TMB Gordia AP Andaki ACR Mendes EL Mota J Silva LR. Triagem da pressão arterial elevada em crianças e adolescentes de Amargosa, Bahia: utilidade de indicadores antropométricos de obesidade. Rev Bras Epidemiol. (2019) 22:e190017. 10.1590/1980-549720190017 30916142

[14] Tu W Eckert GJ Dimeglio LA Yu Z Jung J Pratt JH. Intensified effect of adiposity on blood pressure in overweight and obese children. Hypertension. (2011) 58:818–24. 10.1161/HYPERTENSIONAHA.111.175695 21968752 PMC3433397

[15] Pires A Martins P Pereira AM Marques M Castela E Sena C. Childhood adiposity: being male is a potential cardiovascular risk factor. Eur J Pediatr. (2016) 175:63–9. 10.1007/s00431-015-2599-0 26226893

[16] Bruyndonckx L Hoymans VY Craenenbroeck AHV Vissers DK Vrints CJ Ramet J. Assessment of endothelial dysfunction in childhood obesity and clinical use. Oxid. Med. Cell. Longev. (2013) 2013: 174782. 10.1155/2013/174782 PMC3649697 23691262

[17] Charakida M Jones A Falaschetti E Khan T Finer N Sattar N. Childhood obesity and vascular phenotypes: a population study. J Am Coll Cardiol. (2012) 60:2643–50. 10.1016/j.jacc.2012.08.1017 23177297

[18] Anyaegbu EI Dharnidharka VR. Hypertension in the teenager. Pediatric Clinics. (2014) 61:131–51. 10.1016/j.pcl.2013.09.011 24267462 PMC3947917

[19] Andrade H Pires A Noronha N Amaral ME Lopes L Martins P. Importance of ambulatory blood pressure monitoring in the diagnosis and prognosis of pediatric hypertension. Rev Port Cardiol. (2018) 37:783–9. 10.1016/j.repce.2018.08.005 29871785

[20] De Almeida FA Konigsfeld HP Machado LMO Canadas AF Issa EYO Giordano RH. Assessment of social and economic influences on blood pressure of adolescents in public and private schools: an epidemiological study. J Bras Nefrol. (2011) 33:142–9. 10.1590/S0101-28002011000200005 21789427

[21] Beck CC Lopes AS Pitanga FJG. Anthropometric indicators as predictors of high blood pressure in adolescents. Arq Bras Cardiol. (2011) 96:126–33. 10.1590/S0066-782X2010005000153 21085760

[22] Krakauer NY Krakauer JC. A new body shape index predicts mortality hazard independently of body mass index. PLoS ONE. (2012) 7:e39504. 10.1371/journal.pone.0039504 22815707 PMC3399847

[23] Cureau FV Reichert FF. Anthropometric indicators of obesity as predictors of high blood pressure in adolescents. Rev Bras de Cineantropometria e Desempenho Hum. (2013) 15:338–49. 10.5007/1980-0037.2013v15n3p33830139344

[24] Duncan MJ Mota J Vale S Santos MP Ribeiro JC. Associations between body mass index, waist circumference and body shape index with resting blood pressure in Portuguese adolescents. Ann Hum Biol. (2013) 40:163–7. 10.3109/03014460.2012.752861 23327095

[25] Zhang Y Zhao-Xia W Zun-Hua C Jin-Shan Z. Blood pressure profiles of children and adolescents categorized by waist-to-height ratio cutoffs: study in a large sample in Shandong, China. Blood Press Monit. (2017) 22:143–8. 10.1097/MBP.0000000000000249 28240683

[26] Pelegrini A Silva DAS Lima Silva JMF Grigollo L Petroski EL. Anthropometric indicators of obesity in the prediction of high body fat in adolescents. Rev Paul Pediatr. (2015) 33:56–62. 10.1016/S2359-3482(15)30031-2 25649384 PMC4436957

[27] Zhang Y Wang SR Zhao JS Chu ZH. Truncal pattern of subcutaneous fat distribution is associated with obesity and elevated blood pressure among children and adolescents. Blood Press. (2018) 27:25–31. 10.1080/08037051.2017.1369000 28830245

[28] Dong Y Song Y Zou Z Ma J Dong B Prochaska JJ. Updates to pediatric hypertension guidelines: influence on classification of high blood pressure in children and adolescents. J Hypertens. (2019) 37:297. 10.1097/HJH.0000000000001903 30044314 PMC6365252

[29] Pinto SL Silva RCR Priore SE Assis AMO Pinto EJ. Prevalence of pre-hypertension and arterial hypertension and evaluation of associated factors in children and adolescents in public schools in Salvador, Bahia State, Brazil. Cad Saúde Pública. (2011) 27:1065–75. 10.1590/S0102-311X2011000600004 21710004

[30] Chauí M. Brasil: mito fundador e sociedade autoritária-4^a^ reimpressão. São Paulo: Perseu Abramo (2001). p. 103.

[31] Domingos E Domingues V Júnior RP Caldeira AS Christofaro DGD Casonatto J. Associação entre estado nutricional antropométrico, circunferência de cintura e pressão arterial em adolescentes. Rev Bras Cardiol. (2013) 26:94–9. Available online at: https://www.researchgate.net/publication/239040264_Associacao_entre_estado_nutricional_antropometrico_circunferencia_de_cintura_e_pressao_arterial_em_adolescentes (acessed June 23, 2022).

[32] Moser DC Giuliano ICB Titski ACK Gaya AR. Coelho-e-Silva MJ, Leite N. Anthropometric measures and blood pressure in school children. J Pediatr. (2013) 89:243–9. 10.1016/j.jped.2012.11.006 23684458

[33] Oliveira AV Costa ACPJ Pascoal LM Santos LH Chaves ES Araújo MFM. Correlação entre indicadores antropométricos e pressão arterial de adolescentes. Texto Contexto Enferm. (2014) 23:995–1003. 10.1590/0104-07072014003380013

[34] Cordeiro JP Dalmaso SB Anceschi AS Sá FGS Ferreira LG Cunha MRH. Hipertensão em estudantes da rede pública de Vitória/ES: influência do sobrepeso e obesidade. Rev Bras Med esporte. (2016) 22:59–65. 10.1590/1517-869220162201134305

[35] Wheelock KM Fufaa GD Nelson RG Hanson RL Knowler WC Sinha M. Cardiometabolic risk profile based on body mass index in American Indian children and adolescents. Pediatric obes. (2017) 12:295–303. 10.1111/ijpo.12142 27170264

[36] De Araújo Pinto A Claumann GS Amaral LC Pelegrini A. Prevalência de pressão arterial elevada em adolescentes e associação com indicadores antropométricos. Medicina (Ribeirão Preto). (2017) 50:237–44. 10.11606/issn.2176-7262.v50i4p237-244

[37] Borges LS. Medidas de Acurácia diagnóstica na pesquisa cardiovascular. Int J Cardiovasc Sci. (2016) 29:218–22. 10.1590/1982-0194201600030

[38] PNUD Brasil. Atlas do Desenvolvimento Humano no Brasil (2020). http://www.atlasbrasil.org.br/ranking, (accessed Nov 17, 2021).

[39] Luiz RR Magnanini MMF. A lógica da determinação do tamanho da amostra em investigaçöes epidemiológicas. Cad Saúde Colet (Rio J). (2000) 8:9–28. Available online at: https://pesquisa.bvsalud.org/portal/resource/pt/lil-326604 (acessed June 23, 2022).

[40] Machin D Campbell MJ Tan SB Tan SH. Sample sizes for clinical, laboratory and epidemiology studies. Wiley-Blackwell. (2018). 10.1002/9781118874905

[41] Norton K Olds T Mazza JC Cuesta G Palma M. Antropométrica: un libro de referencia sobre mediciones corporales humanas para la educación en deportes y salud. Rosário: Biomsystem. (2000) 3:102–16.

[42] Chor D. Hipertensão arterial entre funcionários de Banco Estatal no Rio de Janeiro. Hábitos de vida e tratamento Arq Bras Cardiol. (1998) 71:653–60. 10.1590/S0066-782X1998001100003 10347947

[43] Lima TR González-Chica DA Moreno YMF Silva DAS. Healthy lifestyle moderates the relationship between cardiovascular disease with blood pressure, body composition, carotid intima-media thickness, and glycated hemoglobin among adults. Appl. physiol. nutr. metab. (2019) 45:539-46. 10.1139/apnm-2019-0515 31644883

[44] Barroso WKS Rodrigues CIS Bortolotto LA Gomes MAM Brandão AA Feitosa ADM. Diretrizes Brasileiras de Hipertensão Arterial−2020. Arq Bras Cardiol. (2020) 116:516–658. 10.36660/abc.20201238 33909761 PMC9949730

[45] Christofaro DGD Fernandes RA Gerage AM Alves MJ Polito MD Oliveira AR. Validação do monitor de medida de pressão arterial Omron HEM 742 em adolescentes. Arq Bras Cardiol. (2009) 92:10–5. 10.1590/S0066-782X2009000100003 19219259

[46] Stewart A Marfell-Jones M Olds T De Rider H. International standards for anthropometric assessment (ISAK). New Zealand: Lower Hutt. (2011).

[47] Woolcott OO Bergman RN. Relative fat mass as an estimator of whole-body fat percentage among children and adolescents: a cross-sectional study using NHANES. Sci Rep. (2019) 9:1–14. 10.1038/s41598-019-51701-z 31649287 PMC6813362

[48] Valdez R. A simple model-based index of abdominal adiposity. J Clin Epidemiol. (1991) 44:955–6. 10.1016/0895-4356(91)90059-I 1890438

[49] Bergman RN Stefanovski D Buchanan TA Summer AE Reynolds JC Sebring NG. A better index of body adiposity. Obesity. (2011) 19:1083–9. 10.1038/oby.2011.38 21372804 PMC3275633

[50] Brasil Cortes do Critério. Critério de classificação econômica Brasil. Associação Brasileira de Empresas de Pesquisa (ABEP), (2021). Available online at: https://www.abep.org/ (accessed December 8, 2021).

[51] Guedes DP Lopes CC. Validação da versão brasileira do youth risk behavior survey 2007. Rev Saúde Públ. (2010) 44:840–50. 10.1590/S0034-89102010000500009 20877922

[52] World Health Organization. WHO Guidelines on Physical Activity and Sedentary Behavior. Geneva: World Health Organization (2020).

[53] Wilson DMC Nielsen E Ciliska D. Lifestyle assessment: testing the FANTASTIC instrument. Can fam physician. (1984) 30:1863.24897489

[54] Rodriguez Añez CR Reis RS Petroski EL. Versão brasileira do questionário “estilo de vida fantástico”: tradução e validação para adultos jovens. Arq Bras Cardiol. (2008) 91:102–9. 10.1590/S0066-782X2008001400006 18709260

[55] Silva AF Martins PC Gonçalves ECA Farias JM Silva DAS. Prevalence and factors associated with sedentary behavior in the school recess among adolescents. Mot Rev de Educ Fis. (2018) 24:4. 10.1590/s1980-657420180004001422427317

[56] De Lima TR Silva DAS. Association of sleep quality with sociodemographic factors and lifestyle in adolescents from southern Brazil. World J Pediatr. (2018) 14:383–91. 10.1007/s12519-018-0136-8 29536340

[57] Tanner J. Growth at Adolescence: With a General Consideration of the Effects of 524 Hereditary and Environmental Factors Upon Growth and Maturation From Birth to Maturity. Oxford. England: Blackwell Scientific. (1962). p. 526.

[58] Matsudo SMM Matsudo VKR. Self-assessment and physician assessment of sexual maturation in Brazilian boys and girls: concordance and reproducibility. Am J Hum Biol. (1994) 6:451–5. 10.1002/ajhb.1310060406 28548259

[59] Quadros TMB Gordia AP Silva DAS Mota J. Epidemiological survey in schoolchildren: determinants and prevalence of cardiovascular risk factors. Cad Saude Publica. (2016) 32:2. 10.1590/0102-311x00181514 26958824

[60] Swets JA. The relative operating characteristic in psychology: a technique for isolating effects of response bias finds wide use in the study of perception and cognition. Science. (1973) 182:990–1000. 10.1126/science.182.4116.990 17833780

[61] Sopelete MC Mineo JR Silva DAO Leal GS Vidigal LHG Tápia LER. Métodos de análises em estudos sobre diagnóstico. Pesquisa na área biomédica: do planejamento à publicação. (2005):203–14. 10.7476/9788570785237.0009

[62] Aneja A El-Atat F McFarlane SI Sowers JR. Hypertension and obesity. Recent Prog Horm Res. (2004) 59:169–205. 10.1210/rp.59.1.169 14749502

[63] Pinto AA Bim MA Reche LCA Claumann GS Frank R Félden EPG. Indicadores antropométricos como preditores de pressão arterial elevada em adolescentes. Saúde. (2020) 46:2. 10.5902/2236583442557

[64] Moraes MM Veiga GV. Acurácia da gordura corporal e do perímetro da cintura para predizer alterações metabólicas de risco cardiovascular em adolescentes. Arq Bras Endocrinol Metabol. (2014) 58:341–51. 10.1590/0004-2730000002865 24936728

[65] Mazicioglu MM Yalcin BM Ozturk A Ustunbas HB Kurtoglu S. Anthropometric risk factors for elevated blood pressure in adolescents in Turkey aged 11–17. Pediatr Nephrol. (2010) 25:2327–34. 10.1007/s00467-010-1623-x 20721675

[66] Kajale NA Khadilkar AV Chiplonkar SA Khadilkar VV. Body fat indices for identifying risk of hypertension in Indian children. Indian Pediatr. (2014) 51:555–60. 10.1007/s13312-014-0446-4 25031134

[67] World Health Organization (WHO) (2007). Growth reference 5-19 years. Disponível em. Available on at: https://www.who.int/growthref/who2007_bmi_for_age/en/. (accessed January 18, 2022).

[68] Kruger HS Faber M Schutte AE Ellis SM A A proposed cutoff point of waist-to-height ratio for metabolic risk in African township adolescents. Nutrition. (2013) 29:502–7. 10.1016/j.nut.2012.08.009 23274093

[69] Abbaszadeh F Sarafraz N Atrian MK Sadat Z Bagheri A Moravveji A. Anthropometric indices in the prediction of hypertension in female adolescents. Iran Red Crescent Med J. (2017) 19:11. 10.5812/ircmj.14591

[70] Tee JYH Gan WY Lim PY. Comparisons of body mass index, waist circumference, waist-to-height ratio and a body shape index (ABSI) in predicting high blood pressure among Malaysian adolescents: a cross-sectional study. BMJ Open. (2020) 10:e032874. 10.1136/bmjopen-2019-032874 31932391 PMC7044891

[71] Febriana K Nurani N Julia M. Body mass index and waist-to-height ratio cut-offs as predictors of high blood pressure in adolescents. Medical J Indones. (2015) 24:30–5. 10.13181/mji.v24i1.1200

[72] Hsieh SD Yoshinaga H. Abdominal fat distribution and coronary heart disease risk factors in men-waist/height ratio as a simple and useful predictor. Int J Obes Relat Metab Disord. (1995) 19:585–9. 7489031

[73] Ashwell M Hsieh SD. Six reasons why the waist-to-height ratio is a rapid and effective global indicator for health risks of obesity and how its use could simplify the international public health message on obesity. Int J Food Sci Nutr. (2005) 56:303–7. 10.1080/09637480500195066 16236591

[74] Ripka WL Orsso CE Haqq AM Prado CM Ulbricht L Leite N. Validity and accuracy of body fat prediction equations using anthropometrics measurements in adolescents. Eat Weight Disor. (2021) 26:879–86. 10.1007/s40519-020-00918-3 32430885

[75] Slaughter MH Lohman TG Boileau RA Horswill CA Stillman RJ Van Loan MD. Skinfold equations for estimation of body fatness in children and youth. Hum. Biol. (1988):709–23. 3224965

[76] Taylor SA Hergenroeder AC. Waist circumference predicts increased cardiometabolic risk in normal weight adolescent males. Int J Pediatr Obes. (2011) 6:e307–311. 10.3109/17477166.2011.575149 21649469

[77] Bloch KV Klein CH Szklo M Kuschnir MCC Abreu GA Barufaldi LA. ERICA: prevalência de hipertensão arterial e obesidade em adolescentes brasileiros. Rev Saúde Públ. (2016) 50:9. 10.1590/S01518-8787.2016050006685 26910553 PMC4767032

[78] Akobeng AK. Understanding diagnostic tests 3: receiver operating characteristic curves. Acta paediatr. (2007) 96:644–7. 10.1111/j.1651-2227.2006.00178.x 17376185

[79] Fischer JE Bachman LM Jaeschke R A A readers' guide to the interpretation of diagnostic test properties: clinical example of sepsis. Intensive Care Med. (2003) 29:1043–51. 10.1007/s00134-003-1761-8 12734652

[80] Christofaro DGD Andrade SM Fernandes RA Cabrera MAS Ritti-Dias RM. The prevalence of high arterial blood pressure in children and adolescents: a systematic review. Rev bras saúde mater infant. (2011) 11:361–7. 10.1590/S1519-38292011000400002

