# Supplementary material for: Proposition of Cutoff Points for Anthropometric Indicators to Identify High Blood Pressure in Adolescents
Source: Front Nutr. 2022 Jul 18;9:874047. doi: 10.3389/fnut.2022.874047 (PMC9339713; doi:10.3389/fnut.2022.874047)
Supplement: Supplementary file 1 [file Data_Sheet_1.pdf]

Supplementary material 1

Picture 1 - Technical error of intra- and inter-evaluator measurement of the variables in this study

Variables	Median	Mean	Minimum value	Maximum value	Maximum TME allowed*
**Height**					
TME (intra-evaluator)	0.00	0.00	0.00	0.00	1.00
TME (inter-evaluator)	0.03	0.03	0.02	0.03	1.50
**Waist circumference**					
TME (intra-evaluator)	0.03	0.04	0.02	0.33	1.00
TME (inter-evaluator)	0.11	0.12	0.07	0.23	1.50
**Hip circumference**					
TME (intra-evaluator)	0.02	0.02	0.01	0.04	1.00
TME (inter-evaluator)	0.10	0.10	0.06	0.13	1.50
**Triceps skinfold**					
TME (intra-evaluator)	0.18	0.21	0.03	0.40	5.00
TME (inter-evaluator)	1.17	1.25	0.59	2.17	7.50
**Subescapularis skinfold**					
TME (intra-evaluator)	0.16	0.16	0.05	0.31	5.00
TME (inter-evaluator)	0.96	0.94	0.35	1.75	7.50
**Supra iliac skinfold**					
TME (intra-evaluator)	0.18	0.22	0.04	0.66	5.00
TME (inter-evaluator)	1.53	1.56	1.11	2.22	7.50
**Calf sfinfold**					
TME (intra-evaluator)	0.22	0.20	0.10	0.35	5.00
TME (inter-evaluator)	0.95	1.01	0.62	1.54	7.50

Note: TME – Relative Measurement Technical Error – values in percentages.
*Maximum TME allowed for experienced anthropometrists (81).

81 - Pederson D, Gore C. Error en la medición antropométrica. In: Norton K, Olds T, editors. Antropométrica. Argentina: Biosystem Servicio Educativo, (2000).
