# Supplementary material for: Proposition of Cutoff Points for Anthropometric Indicators to Identify High Blood Pressure in Adolescents
Source: Front Nutr. 2022 Jul 18;9:874047. doi: 10.3389/fnut.2022.874047 (PMC9339713; doi:10.3389/fnut.2022.874047)
Supplement: Supplementary file 2 [file Data_Sheet_2.pdf]

SUPPLEMENTARY FILE 2

Standardization of anthropometric measurements performed in the present study according to Stewart et al. (2011):

46 - Stewart A, Marfell-Jones M, Olds T, De Rider H. International standards for anthropometric assessment (ISAK). New Zealand: Lower Hutt, (2011).

Body mass:
- Subject position: Anthropometric position
- Method: The subject stands on the centre of the scale without support and with the weight distributed evenly on both feet.

Height:
- Subject position: Anthropometric position but with feet together the back in contact with the stadiometer and the head in the Frankfort plane.
- Equipment: A calibrated stadiometer on hard and level ground.

SKINFOLD MEASUREMENTS:
Triceps
- Subject position: Anthropometric position
- Equipment: A calibrated skinfold caliper
- Method: Point on the posterior aspect of the arm, on the midline at the level of the radial acromial point

Subscapular
- Subject position: Anthropometric position
- Equipment: A calibrated skinfold caliper
- Method: 2 cm below the inferior angle of the scapula, along a line extending laterally and obliquely at an angle of 45°

Iliac crest
- Subject position: Anthropometric position – on the right side of the subject
- Equipment: A calibrated skinfold caliper
- Method: The site of the center of the skinfold that forms just above the iliocrystal point

Calf
- Subject position: Anthropometric position –in front of the medial side of the right leg
- Equipment: A calibrated skinfold caliper
- Method: the skinfold is measured parallel to the longitudinal axis of the leg in the medial region with the greatest perimeter of the calf

GIRTHS:
Waist
- Subject position: Anthropometric position – with forearms crossed over the thorax
- Equipment: A calibrated anthropometric tape.
- Method: narrowest point between the lower costal margin (10th rib) and the upper part of the iliac crest

Hips

- Subject position: Anthropometric position with forearms crossed over the thorax, gluteal muscles relaxed and with the feet together
- Equipment: A calibrated anthropometric tape.
- Method: measured at the level of greatest posterior protuberance of the glutes, perpendicular to the longitudinal axis of the trunk
